# Dietary macronutrients do not differently affect postprandial vascular endothelial function in apparently healthy overweight and slightly obese men

**DOI:** 10.1007/s00394-020-02340-y

**Published:** 2020-07-29

**Authors:** Ellen T. H. C. Smeets, Ronald P. Mensink, Peter J. Joris

**Affiliations:** Department of Nutrition and Movement Sciences, NUTRIM School for Nutrition and Translational Research in Metabolism, Maastricht University Medical Center, PO Box 616, 6200 MD Maastricht, The Netherlands

**Keywords:** Flow-mediated vasodilation, Dietary macronutrients, Postprandial, Endothelial function, Nitric oxide

## Abstract

**Purpose:**

Well-designed trials comparing side-by-side effects of macronutrients on postprandial endothelial function are missing. Therefore, we investigated under well-controlled and isocaloric condition effects of fat, carbohydrates, and protein on postprandial endothelial function as assessed by brachial artery flow-mediated vasodilation (FMD), an important non-invasive technique to assess endothelial function.

**Methods:**

Eighteen apparently healthy overweight and slightly obese men (BMI 26.0–35.0 kg/m^2^) completed this randomized, double-blinded, cross-over trial. The study consisted of three test days each separated by a wash-out period of at least 1 week. After an overnight fast, men received an isocaloric meal providing 3987 kJ (953 kcal) that was either high in dietary fat (En% fat [F]/carbohydrates [C]/protein [P]: 52.3, 39.2, 8.0), carbohydrates (En% F/C/P: 9.6, 81.5, 8.6), or protein (En% F/C/P: 10.6, 51.5, 36.9). Fasting and 2-h postprandial FMD responses were measured.

**Results:**

A postprandial decrease of 1.2% point in FMD was observed after the high-protein meal (*P* = 0.015). However, postprandial changes did not differ between meals (*P* = 0.45). An increase in baseline brachial artery diameters was observed after the high-protein meal (*P* < 0.001) and changes differed between meals (*P* = 0.020). A meal*time interaction was found for plasma glucose concentrations, with the most pronounced increases after the high-carbohydrate meal at T15, T30, T60, and T90 (*P* < 0.05)*.* A significant time and meal (*P* < 0.001), but no time*meal effect (*P* = 0.06) was found for serum insulin concentrations. Increases in serum triacylglycerol concentrations did not differ between meals (*P* = 0.014).

**Conclusion:**

Macronutrients did not differently affect postprandial endothelial function in apparently healthy overweight and slightly obese men.

**Trial registration:**

Trial registration number (ClinicalTrials.gov) NCT03139890 in May 2017

**Electronic supplementary material:**

The online version of this article (10.1007/s00394-020-02340-y) contains supplementary material, which is available to authorized users.

## Introduction

An important mechanism in the development of cardiovascular disease (CVD), the leading cause of death worldwide, is a reduced nitric oxide (NO) bioavailability, which causes impaired function of the endothelial vascular cell layer [1–3]. NO is a key determinant of endothelial function, since it exerts important vasodilatory, anti-inflammatory, antithrombotic, anti-proliferative, and anti-adhesive effects [2, 4]. It has been hypothesized that meal ingestion as well as meal composition may change NO bioavailability [5–7]. Since we spend most time of the day in the postprandial state, repeated reductions in NO bioavailability caused by meal consumption may thus contribute to the development of CVD [8].

An important non-invasive method to assess vascular NO-dependent endothelial function is brachial artery flow-mediated vasodilation (FMD). It has been estimated that an increase of 1% point (PP) in fasting FMD is associated with a reduction of 8–13% in overall CVD risk [9, 10]. Unfortunately, studies on the effects of meal intake on postprandial FMD are not consistent and a recent meta-analysis reported an overall decrease in postprandial FMD, but heterogeneity in effects between the studies was high [6]. Most studies in healthy and overweight individuals observed a decrease in FMD after consumption of a high-fat meal [11–13]. In contrast, an significant increase in postprandial FMD was observed after consumption of a high-carbohydrate, high-fiber meal, while other studies observed a decrease in FMD after an oral glucose load [14–16]. Effects of protein intake on FMD have been studied less, although Westphal et al. [17] observed that a postprandial impairment in FMD was counteracted by adding proteins to a high-fat challenge. A possible explanation for the observed differences in postprandial FMD responses between studies may thus be the macronutrient composition of meals.

Effects of the dietary macronutrients (i.e. fat, carbohydrates, and protein) on FMD during the postprandial phase under well-controlled and isocaloric conditions have not been studied side-by-side before. Therefore, the aim of this study was to compare in apparently healthy overweight and slightly obese men effects of a mixed-meal high in fat, carbohydrates or protein on postprandial changes in endothelial function, as assessed by FMD.

## Materials and methods

### Study population

Twenty apparently healthy overweight and slightly obese men were recruited through advertisements in local newspapers, flyers in university and city buildings, or among participants who had participated in earlier studies. Important inclusion criteria were: aged between 18 and 70 years, body mass index (BMI) between 25 and 35 kg/m^2^, no smoking or smoking cessation > 1 year, stable body weight (weight gain or loss < 3 kg within the previous 3 months), no use of anti-hypertensive medication or drugs known to affect serum lipid or plasma glucose metabolism, no diabetes or active CVD, and no participation in another trial during the past 30 days. All participants gave written informed consent before entering the study. The study was performed in accordance with the ethical guidelines of the 1975 Declaration of Helsinki. The study was approved by the Medical Ethical Committee of the University Hospital Maastricht/Maastricht University (METC azM/UM), and registered at ClinicalTrials.gov in May 2017 as NCT03139890.

### Study design

A randomized, double-blind, cross-over study was performed. Each participant took part in three test days, each separated by a wash-out period of at least 1 week. On the 2 days preceding each postprandial test day, participants were asked not to perform any strenuous physical activity. They were also not allowed to consume alcohol on the day preceding each test day. Throughout the whole study period, participants were asked not to change their habitual food intake or physical activity levels, which both may affect endothelial function [18, 19].

To standardize measurements, participants came to the Metabolic Research Unit Maastricht by car or by public transport after an overnight fast (from 20.00 h). After insertion of an intravenous cannula, the participant had to rest for 15 min in supine position after which vascular measurements were performed. After completion, a fasting venous blood sample (T0) was collected. Thereafter, the participants had to consume either a high-fat, high-carbohydrate or high-protein blended meal according to a protocol in which each 1/3rd of the meal had to be consumed within 1 min with 2 min breaks. If a subject could not consume 1/3rd within 1 min, the break between consumption periods was shortened accordingly. Subsequent blood samples were collected 15 min (T15), 30 min (T3), 45 min (T45), 60 min (T60), 90 min (T90), 120 min (T120), 180 min (T180), and 240 min (T240) after meal consumption. Immediately after collection of the blood sample at T120, vascular measurements were repeated.

### Meals

Meals were freshly prepared by an independent person on the morning of a test day. All three meals provided 953 kilocalories. The composition of the meals is shown in Table 1. The high-fat meal provided 52.3 En% from fat, 39.2 En% from carbohydrates, and 8.0 En% from protein. For the high-carbohydrate meal, these values were, respectively, 9.6 En%, 81.5, and 8.6 En% and for the high-protein meal 10.6 En%, 51.5 En%, and 36.9 En%. Participants also received glasses of water to correct for differences in weight. The total volume of all three meals, including the glasses of water, was 730 mL.

**Table 1:** Baseline characteristics of the overweight and slightly obese men who completed the study

	Study participants (*n* = 18)
Age (years)	65 (50.8–67.0) and 27–70^a^
BMI (kg/m^2^)	30.5 ± 2.9
Men with overweight/obesity (%)	56/44
Fasting serum total cholesterol (mmol/L)	5.32 ± 0.97
Fasting serum HDL cholesterol (mmol/L)^b^	1.19 ± 0.26
Fasting serum LDL cholesterol (mmol/L)^b^	3.45 ± 0.75
Fasting serum TAG (mmol/L)	1.27 ± 0.47
Fasting plasma glucose (mmol/L)	5.67 ± 0.49
Systolic BP (mmHg)	136.3 ± 11.0
Diastolic BP (mmHg)	86.0 ± 6.7
Heart rate (BPM)	67.6 ± 10.3

Values are means ± SD; obtained during screening visit

*TAG* triacylglycerol, *HDL* high-density lipoprotein, *LDL* low-density lipoprotein, *BP* blood pressure, *BPM* beats per minute

^a^Median (± IQR) and range

^b^Average concentrations from the three different test days

### Anthropometric and vascular measurements

Height was measured during the screening visit using a wall-mounted stadiometer. Before the start of each postprandial test, body weight was measured without shoes and heavy clothing. Furthermore, waist and hip circumferences were measured.

After an acclimatization period of at least 15 min in supine position, office brachial systolic blood pressure (SBP), diastolic blood pressure (DBP), and heart rate (HR) were measured in supine position before the start of the vascular measurements using a semi-continuous blood pressure monitoring device (Omron M7 Intelli^TM^sense, Omron, Hoofddorp, The Netherlands). The first measurement was discarded and the average of the last three measurements was reported. Vascular measurements were performed in a quiet and darkened room with a stable room temperature at 22 °C. All measurements were performed in a fasting state and two hours after the meal by the same sonographer (ES).

For the first 11 participants, FMD measurements were made by a Hewlett-Packard echo-Doppler device (Sonos 5500) using a 7.5-MHz transducer, whereas a MyLab^TM^Gamma (Esaote) with a 13–4 MHz linear transducer was used for the last 9 study participants due to the limited availability of the first echo-Doppler device during the second part of the study. The brachial artery above the elbow was scanned in longitudinal direction to ensure proper visualization of the brachial artery before the start and during the measurement. After a 3-min reference period, the pneumatic cuff placed distal to the ultrasound probe was inflated to 200 mmHg, causing distal hypoxia. After 5 min of inflation, the cuff was deflated to induce forearm reactive hyperemia causing an increase in brachial artery diameter. The echo images were processed automatically to determine the diameter profiles over the entire 13 min measurement using a custom-written Matlab program (MyFMD 2014, Prof. APG Hoeks, Department of Biomedical Engineering, Maastricht University, Maastricht, The Netherlands). The FMD was calculated as the percentage change in post occlusion peak brachial artery diameter relative to the baseline brachial artery diameter.

Pulse wave analysis (PWA) of the radial artery near the wrist was performed, in triplicate, with a tonometer (SphygmoCor V9, AtCor Medical Inc, Itasca, Illinois, USA). A validated transfer function was used to derive the central arterial waveform from the peripheral waveform. Central augmentation indices, which are a measure of wave reflection, were defined as the difference between the first and second peak of the arterial waveform, expressed as percentage of the pulse pressure and adjusted for heart rate (CAIxHR75).

### Biochemical analyses

Blood was sampled in 4 mL tubes containing sodium fluoride and Na_2_EDTA (Becton Dickinson, Erembodegem, Belgium) and 6-mL EDTA tubes (Becton Dickinson). To obtain plasma, the tubes were centrifuged at 1300×*g* for 15 min at 4 °C immediately after sampling. Furthermore, blood was collected in 6-mL serum STT-II advance tubes (Becton Dickinson) and placed for at least 30 min at 21 °C allowing the blood to clot. After clotting, the tubes were centrifuged at 1300×*g* for 15 min at 21 °C to obtain serum. Following centrifugation, plasma and serum were immediately portioned into aliquots, frozen into liquid nitrogen and stored at − 80 °C until analysis at the end of the study.

Plasma glucose (Glucose HK CP, Horiba ABX, Montpellier, France) and plasma-free fatty acid (FFA; NEFA-HR, HUIJFILM Wako Diagnostics U.S.A. Corp. Mountain View, CA, USA) concentrations, were measured in NaF plasma at all time points. Also, serum insulin (human insulin specific RIA kit; Millipore, Billerica, MA, USA) concentrations were measured at all time points. Serum triacylglycerol (TAG; GPO Trinder, Sigma-Aldrich Corp., St. Louis, MO, USA) with correction for free glycerol was measured at T0, T30, T60, T120, T180 and T240. Serum total cholesterol (CHOD-PAP method; Roche Diagnostic Systems), high-density lipoprotein cholesterol (HDL cholesterol; precipitation method; Roche Diagnostics, Mannheim, Germany), and plasma high-sensitivity C-reactive protein (hsCRP; CRP CP, Horiba ABX, Montpellier, France) were only measured at T0. Fasting LDL-cholesterol levels were calculated using the Friedewald formula [20].

### Statistical analyses

All data are presented as means and standard deviations (SD), unless stated otherwise. Before the start of the study, it was calculated that 18 participants were needed to detect a true treatment difference of 1.75% point in FMD with an intra-subject variability of 2.3% point, which we observed in our previous study [21], when an alpha of 0.05 and power of 80% were used.

A repeated-measures ANOVA was performed to test for differences in fasting values between test days. To test for significant differences between fasting and postprandial values for all vascular measurements, a paired Student’s *t* test was performed. Separate analyses for each meal on the absolute plasma glucose, serum insulin, FFA, and TAG concentrations were performed to analyze differences between fasting and postprandial concentrations using linear mixed models. Differences in changes between the three test days in FMD, blood pressure, and PWA were analyzed using linear mixed models with period as fixed factor. The effect of time, meal and interaction [time*meal] on plasma glucose, and serum triacylglycerol, insulin and free fatty acid changes from fasting values were analyzed with linear mixed models. If the interaction term was not statistically significant, it was omitted from the model. Post hoc tests with Bonferroni corrections were performed when the interaction term, or factor meal and/or time were statistically significant. Relations between changes in vascular outcomes and incremental area under the curves (iAUC) for glucose and insulin was examined by Pearson correlation coefficients. The iAUC was calculated with the trapezoidal rule [22]. The level of significance was set at *P* < 0.05. All analyses were performed using SPSS 23.0 software for Mac (SPSS Inc. Chicago, IL, USA).

## Results

### Study participants

Of the 23 participants that were screened, three men were excluded due to fasting serum TAG concentrations above 2.2 mmol/L. Of the 20 men enrolled, 18 completed all three test days. Two subjects dropped-out, because of personal reasons (Supplemental Fig. 1). The median age of the men who completed the trial was 65 years and their average BMI was 30.5 ± 2.9 kg/m^2^. Baseline characteristics are shown in Table 1.

### Vascular function

**Fig. 1:**
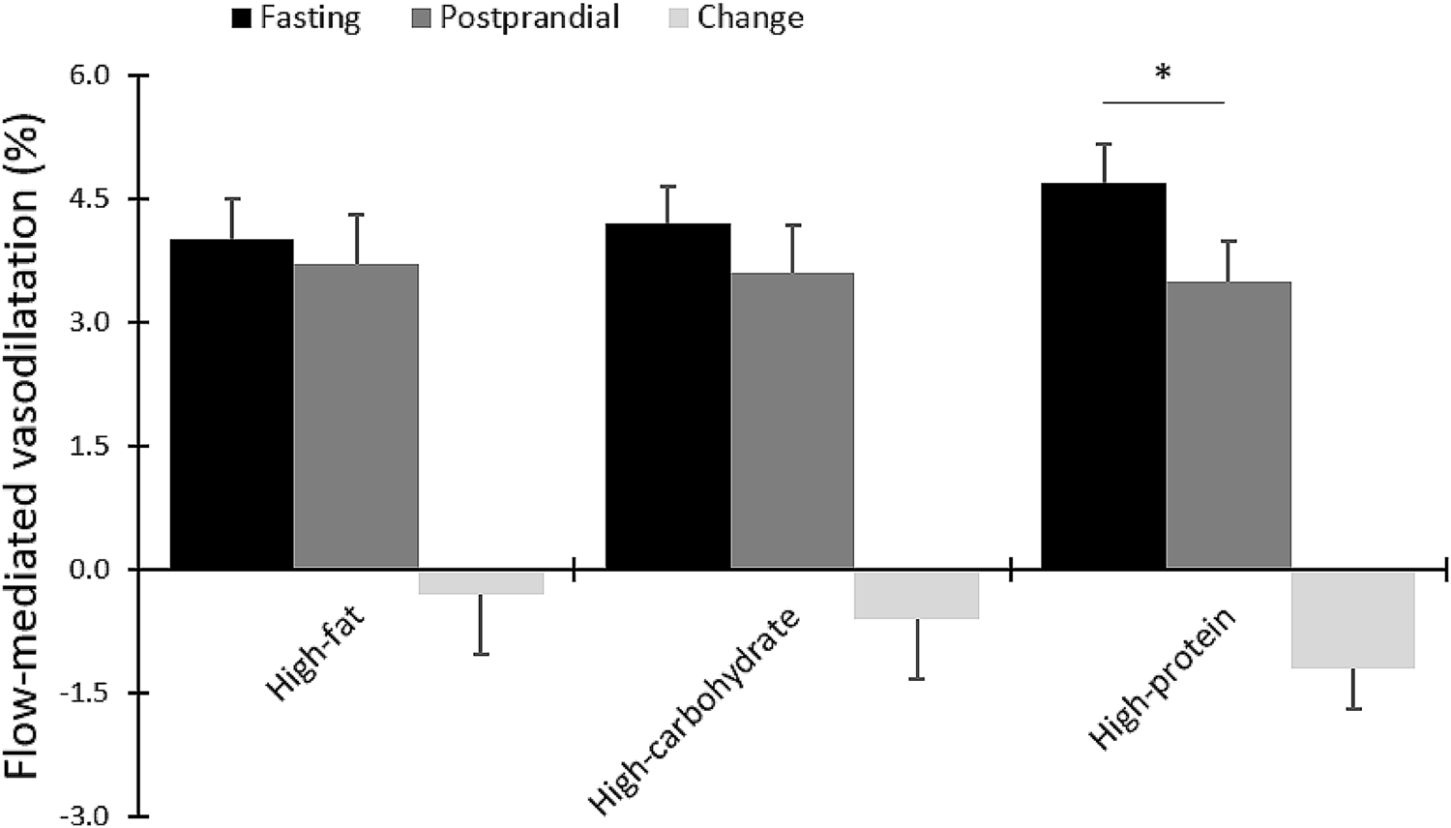
Mean flow-mediated vasodilatation (± SEM) before and after the high-fat, high-carbohydrate and high-protein meals (*n* = 18). * Significantly different from fasting values, *P* < 0.05 (paired Student’s *t* test)

The observed intra-subject variability in this study was below the 2.3% point. Fasting FMD values (*P* = 0.20) and brachial artery diameters (*P* = 0.58) did not differ between meals. FMD decreased after consumption of the high-protein meal (*P* = 0.015), but no changes were observed after consumption of the high-fat or high-carbohydrate meals (*P* = 0.94 and *P* = 0.36, respectively). However, postprandial changes in FMD did not differ between meals (*P* = 0.45) (Fig. 1). Results for FMD did not differ between overweight and obese men (results not shown). The brachial artery diameter was increased after the high-protein meal (*P* < 0.001) and different between meals (*P* = 0.020). Post hoc analyses showed that the baseline brachial artery diameter was increased after the high-protein meal as compared with the high-fat meal (*P* = 0.018).

As indicated in Table 2, fasting SBP and DBP values did not differ between meals (*P* = 0.25 and *P* = 0.12, respectively). Fasting HR, however, was significantly different between meals (*P* = 0.020) and a trend for a higher HR was observed before the high-carbohydrate meal compared with the high-fat and high-protein meals (*P* = 0.050 and *P* = 0.038, respectively). Postprandial changes in brachial SBP and DBP were different between meals (*P* = 0.020 and *P* = 0.002, respectively), but changes in heart rate did not differ (*P* = 0.16). A significant difference between the high-fat and high-carbohydrate meal was observed for SBP and DBP (*P* = 0.009 and *P* = 0.001, respectively; Table 2). SBP and DBP significantly decreased after consumption of the high-carbohydrate meal (*P* = 0.004 and *P* < 0.001, respectively). Moreover, DBP, but not SBP, decreased after consumption of the high-protein meal (*P* = 0.010). Due to technical issues CAIxHR75 measurements were available for seventeen participants for the high-fat meal, sixteen participants for the high-carbohydrate meal and 18 participants for the high-protein meal. CAIxHR75 was significantly reduced after consumption of the three meals (*P* < 0.001 for all meals), but effects did not differ between meals (*P* = 0.118).

**Table 2:** Blood pressure and vascular stiffness measurements before and after consumption of the high-fat, high-carbohydrate or high-protein meal in overweight and slightly obese men

	High fat			High carbohydrate			High protein		
	Fasting	2 h	Delta	Fasting	2 h	Delta	Fasting	2 h	Delta
Baseline artery diameter (mm)	4.9 ± 0.6	4.9 ± 0.6	0.0 ± 0.2	4.8 ± 0.5	4.9 ± 0.6	0.1 ± 0.2	4.8 ± 0.5	4.9 ± 0.5^a^	0.2 ± 0.1^c^
CAIxHR75 (%)^d^	14.8 ± 11.0	10.1 ± 13.0^c^	− 4.7 ± 3.0	14.5 ± 12.3	8.2 ± 10.3^a^	-6.3 ± 5.4	15.0 ± 11.0	7.5 ± 11.8^a^	− 7.5 ± 4.5
SBP (mmHg)	131.9 ± 13.5	132.9 ± 15.6	0.9 ± 6.8^b^	134.2 ± 13.8	129.8 ± 12.6^a^	-4.4 ± 5.7^c^	132.6 ± 14.0	132.2 ± 13.8	− 0.4 ± 6.6
DBP (mmHg)	81.0 ± 7.4	80.4 ± 7.9	− 0.6 ± 4.4^b^	82.8 ± 7.5	76.7 ± 6.0^a^	-6.1 ± 4.0^c^	81.2 ± 7.9	78.0 ± 8.0^a^	− 3.2 ± 4.7
HR (BPM)	58 ± 7	59 ± 7	0.4 ± 2.4	60 ± 7	61 ± 7	0.9 ± 2.5	58 ± 7	61 ± 8	2.2 ± 4.9

Values are presented as means ± SD

*SBP* systolic blood pressure, *DBP* diastolic blood pressure, *HR* heart rate, *CAIxHR75* central augmentation index adjusted for heart rate. *n* = 18 for all analyses

^a^Significantly different as compared with fasting values of the same intervention, *P* < 0.05

^b^Significantly different as compared with the high-carbohydrate meal, *P* < 0.05

^c^Significantly different as compared with the high-fat meal, *P* < 0.05

^d^High fat: *n* = 17; high carbohydrate: *n* = 16; high protein: *n* = 18

### Postprandial lipemia and glycemia

**Fig. 2:**
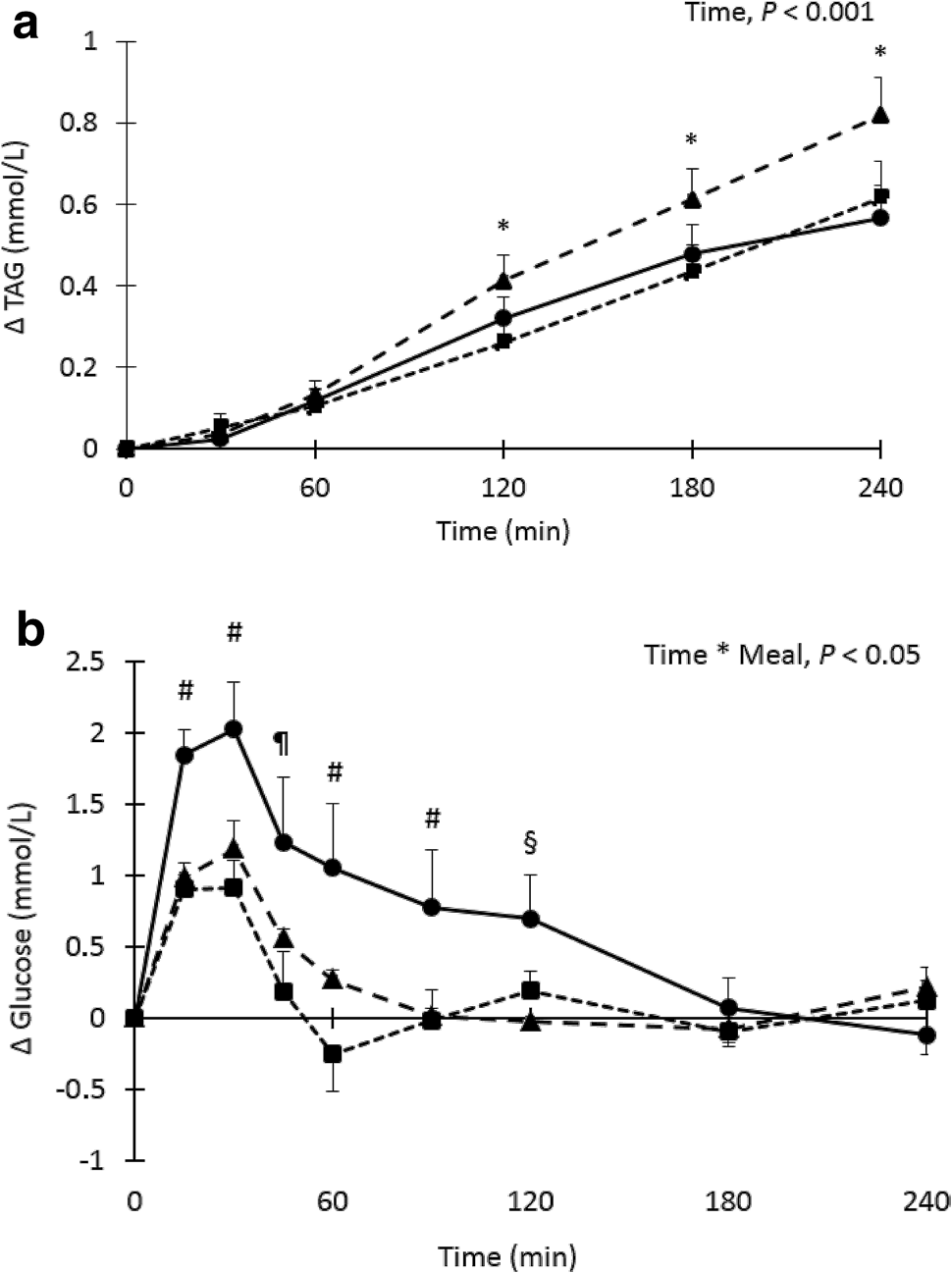

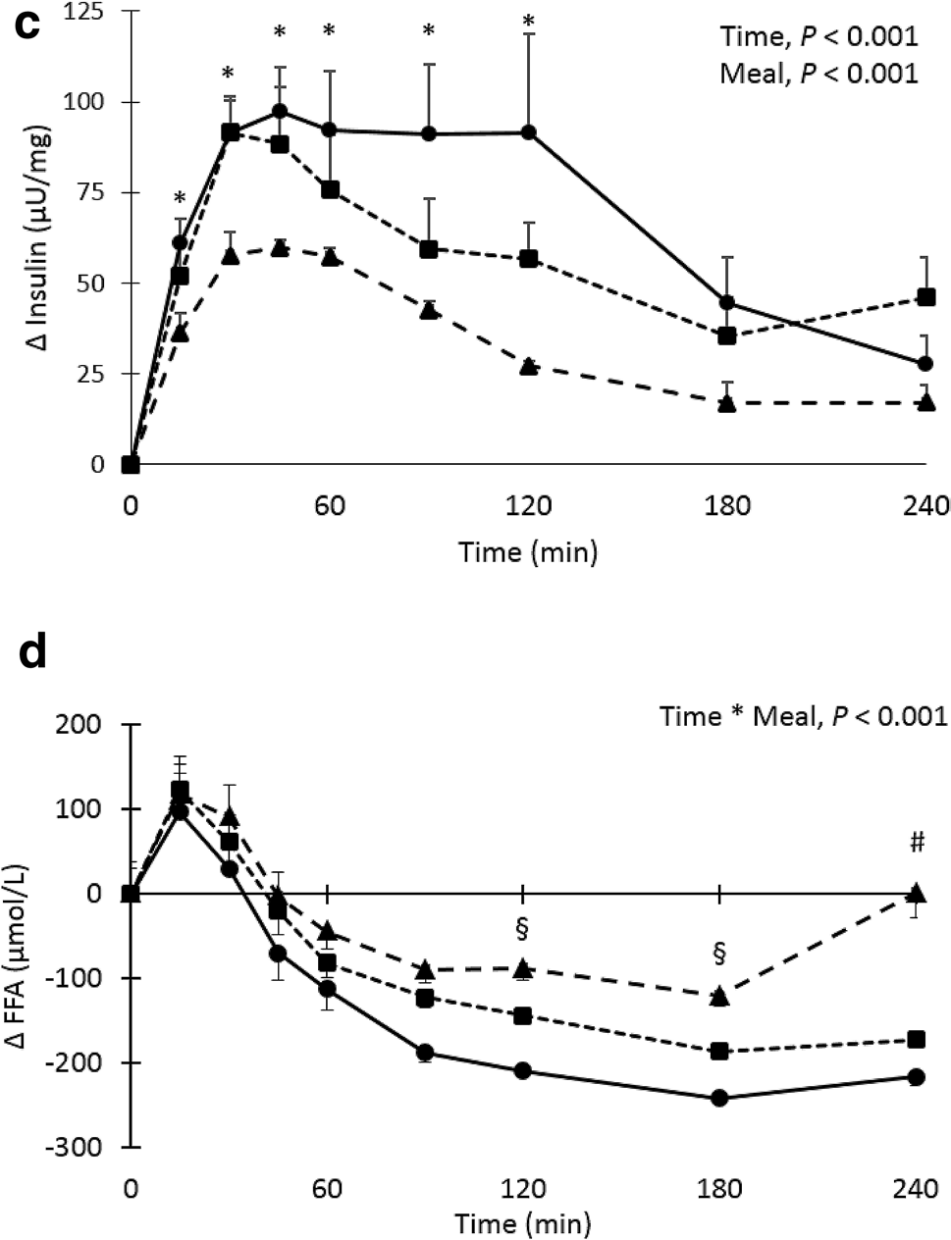
**a**–**d** Mean changes (± SEM) in serum triacylglycerol (TAG), plasma glucose, serum insulin and serum free fatty acids (FFA) concentrations after consumption of either the high-fat (closed triangle), high-carbohydrate (closed circle) and high-protein (closed square) meal in a randomized cross-over study in overweight and slightly obese men (*n* = 18). Data were analyzed using linear mixed models. ^*^After Bonferroni’s correction significantly different from fasting values, *P* < 0.05. ^#^After Bonferroni’s correction, significantly different from the other two meals. ^§^After Bonferroni’s correction, significantly difference between high-carbohydrate and high-fat meal. ^¶^After Bonferroni’s correction, significantly different between high-carbohydrate and high-protein meal

Fasting total cholesterol, HDL cholesterol, LDL cholesterol, and hsCRP concentrations did not differ between meals (Supplemental Table 2). Fasting serum TAG concentrations were also comparable (*P* = 0.35). After meal consumption, postprandial TAG concentrations were significantly increased at T120, T180 and T240 from fasting concentrations (*P* < 0.001 at all time points, Fig. 2a), but there were no significant differences between meals (*P* = 0.14).

Fasting plasma glucose concentrations were comparable between the three meals (*P* = 0.49). However, a significant time*meal (*P* = 0.038) effect was found and plasma glucose concentrations were significantly higher after consumption of the high-carbohydrate meal as compared with the high-fat and high-protein meals at time points T15, T30, T60, and T90 (*P* < 0.05 for all time points). Glucose concentrations were only different between the high-carbohydrate and the high-protein meals at T45 (*P* = 0.001), while a difference between the high-carbohydrate and high-fat meals was only observed 120 min after meal consumption (*P* = 0.015, Fig. 2b).

Fasting serum insulin concentrations did significantly differ between the three meals (*P* = 0.025) and was lower on the high-carbohydrate meals as compared with the high-protein meal (11.05 and 13.3 µU/mg, respectively, *P* = 0.031). A significant time (*P* < 0.001) and meal (*P* < 0.001) effect, but no time*meal effect (*P* = 0.06) was found. Serum insulin concentrations were significantly higher after consumption of the high-carbohydrate (*P* < 0.001) and the high-protein (*P* = 0.001) meals compared with the high-fat meal. After meal consumption, postprandial serum insulin concentrations were significantly higher at T15 to T90 from fasting concentrations (*P* < 0.05 at all time points, Fig. 2c).

Fasting plasma FFA concentrations were comparable between meals (*P* = 0.22). A significant time*meal effect (*P* < 0.001) was found for postprandial plasma FFA concentrations and values were significantly higher 240 min after consumption of the high-carbohydrate meal as compared with the high-fat and high-protein meals (*P* < 0.001). At T120 and T180 FFA, values were only significantly higher after the high-carbohydrate compared with the high-fat meal (*P* = 0.022 and *P* = 0.023, respectively, Fig. 2d).

No correlations were found between the changes in FMD, CAIxHR75, DBP, SBP, and HR with the iAUCs of glucose, insulin, and TAG.

## Discussion

In this well-controlled randomized study with apparently healthy overweight and slightly obese men, the acute intake of a high-protein meal decreased FMD during the postprandial phase, while no effects of a high-carbohydrate or high-fat meals were observed. However, differences between the meals did not reach statistical significance. In addition, the baseline brachial artery diameter was significantly increased after the high-protein meal.

Thom and colleagues suggested in their recent meta-analysis involving 78 acute studies that the intake of a single meal significantly decreased postprandial FMD values after 1, 2 and 3 h in most, but not in all studies [6]. Heterogeneity in outcomes between studies was observed. Although not assessed, a possible explanation for this heterogeneity may relate to differences in macronutrient composition of the meals. However, effects of dietary macronutrients on FMD have not been compared side-by-side before.

Earlier, we have shown that in overweight and slightly obese men, FMD was decreased 2-h after intake of a high-fat meal [21]. Borucki et al. [23] have also reported that consumption of a high-fat meal in healthy subjects significantly decreased FMD from fasting values after 2 h, which was accompanied by a significant increase in postprandial serum TAG concentrations. There are at least two physiological mechanisms to explain this postprandial impairment. First, postprandial lipemia, caused by high-fat meal intake, induces oxidative stress, which in turn results in impaired endothelial function by reducing NO availability [24]. Second, impaired endothelial function might be caused by direct adverse effects of triglyceride-rich lipoprotein particles and their remnants on the endothelial vascular cell layer [25]. In our study, serum TAG concentrations were significantly increased 2 h after the intake of the high-fat meal, but increases in TAG concentrations did not differ between meals (*P* = 0.14). Despite these increases, postprandial FMD did not significantly decrease after consumption of the high-fat meal. However, the highest serum TAG concentrations were observed 4-h post-consumption of the high-fat meal, whereas effects on FMD were assessed after 2 h. Of note, in a similar study, we found no decrease in 4-h postprandial FMD after a high-fat meal involving a similar research population [26, 27]. Therefore, it is possible that a high-fat meal per se has no substantial effects on postprandial FMD and that other mechanisms are involved. Finally, the relatively low fasting FMD values of our population (3.8%) are also not a likely explanation, as in our previous study [21], we observed a postprandial decrease in FMD after a high-fat meal with similar fasting FMD values. A difference with that study [21] is that we now used liquid formula meals, whereas in the earlier study, a high-fat muffin (i.e. a solid food) was used. However, Borucki et al. also used a liquid fatty meal, but the amount of fat provided in their study (~ 70 g) was higher than in this study (~ 55 g).

Another mechanism by which macronutrients may affect postprandial FMD responses is through hyperglycemia. Hyperglycemia may limit the bioavailability of l-arginine, thereby limiting the substrate availability for endogenous endothelial NO production by the enzyme endothelial nitric oxide synthase (eNOS) [8]. Moreover, studies have shown that hyperglycemia increases plasma asymmetrical dimethylarginine (ADMA) concentrations, which inhibit eNOS thereby further reducing the NO bioavailability [28, 29]. Although we observed that the high-carbohydrate meal significantly increased plasma glucose concentrations compared with the high-fat and high-protein meals, we did not observe a postprandial impairment of FMD response after the high-carbohydrate meal. In contrast, Kawano et al. [16] reported a significant decrease in 2-h FMD after an oral glucose load in subjects with impaired glucose tolerance and patients with type II diabetes mellitus, but not in normal glucose tolerant subjects that obviously had lower postprandial increases in plasma glucose concentrations (6.4 mmol/L vs. 1.3 mmol/L, respectively). Therefore, the study population may be another important determinant explaining observed differences in postprandial FMD responses. Another difference is that we used a high-carbohydrate mixed meal, whereas other studies that observed postprandial decreases in FMD through hyperglycemia investigated the effects of an oral glucose load (i.e. 100 En% carbohydrates) [15, 16, 30]. Therefore, a possible explanation is thus that a high-carbohydrate meal has no effect on postprandial FMD in apparently healthy subjects and possible effects are only observed after an oral glucose load.

Although effects of high-protein meals have been studied less, Westphal et al. [17] found that addition of soy or casein proteins to a high-fat meal counteracted the fat-induced impairment in FMD. Despite this observation, we did not find a postprandial difference in FMD after the high-protein meal compared with the high-fat meal. Unexpectedly, the baseline brachial artery diameter was increased after the high-protein meal and these changes were also different between the meals. Atkinson and Batterham have stated that baseline diameter is an important confounder of the FMD [31]. Thus, the observed increase in postprandial baseline diameter after high-protein intake might explain this postprandial decrease in FMD. Observed postprandial differences in baseline brachial artery diameters could not be explained by differences in serum TAG concentrations, as the high-protein meal caused similar increases compared with the high-fat and high-carbohydrate meals. Furthermore, the high-protein meal caused similar increases in plasma glucose concentrations compared with the high-fat meal, but not compared with the high-carbohydrate meal. Another possible mechanism behind an increase in baseline brachial artery diameter might be postprandial insulin concentrations, as insulin exerts vasodilating actions through activation of NO [32]. However, both the high-carbohydrate and high-protein meal significantly increased serum insulin levels compared with the high-fat meal, but only the high-protein meal showed a significantly increase in postprandial brachial artery diameters. Finally, a possible explanation is that our findings on baseline brachial diameters are due to chance and that a high-protein meal has no substantial effect on postprandial FMD.

A significant decrease in CAIxHR75 was observed, but postprandial decreases did not differ between meals. This indicates similar postprandial decreases in the tone of peripheral resistance arteries between the meals. Finally, significant differences between meals were found for postprandial changes in brachial SBP and DBP. Post hoc analyses revealed a significant difference between the high-fat and high-carbohydrate meals. A significant decrease in postprandial SBP after the high-carbohydrate meal was found, whereas postprandial DBP significantly decreased after the high-carbohydrate and high-protein meals. Since only the high-protein meal significantly increased postprandial baseline brachial artery diameters, the blood pressure results cannot be explained by changes in brachial diameters. Moreover, decreases in blood pressure, as well as CAIxHR75, were in our study not related to postprandial increases in serum insulin concentrations.

This study has some potential limitations. First, we included only men. Although this excludes possible variations in the outcomes due to sex effects, it reduces the external validity of the results. Second, this study was adequately powered to find changes in our primary outcome parameter, our sample size was too small to perform subgroup analyses. Finally, FMD was assessed using two different devices. However, all measurements for a particular participant were performed using the same device. Important strengths of this study include the double-blind design, while effects on FMD, which is an important, widely used, non-invasive measure to assess endothelial function [33], were examined under well-controlled and isocaloric conditions.

In conclusion, our study shows that the high-fat, high-carbohydrate and high-protein meals did not differently affect 2-h postprandial FMD responses in overweight and slightly obese men. These findings could not be explained by postprandial differences in TAG, glucose, or insulin responses. If differences in effects between our study and previous studies relate to differences in meal consistency or study population warrant further study. Finally, potential effects on baseline brachial artery diameters should be taken into account when interpreting results on endothelial function, as assessed by FMD.

## Electronic supplementary material

Below is the link to the electronic supplementary material.

Supplementary file1 (DOCX 27 kb)

Supplementary file2 (PDF 183 kb)

Supplementary file3 (PDF 197 kb)
