# Supplementary material for: Dietary macronutrients do not differently affect postprandial vascular endothelial function in apparently healthy overweight and slightly obese men
Source: Eur J Nutr. 2020 Jul 29;60(3):1443–51. doi: 10.1007/s00394-020-02340-y (PMC7987601; doi:10.1007/s00394-020-02340-y)
Supplement: Supplementary file 1 — Supplementary file1 (DOCX 27 kb) [file 394_2020_2340_MOESM1_ESM.docx]

**Dietary macronutrients do not differently affect postprandial vascular endothelial function in apparently healthy overweight and slightly obese men**

European Journal of Nutrition

Ellen T.H.C. Smeets^a^, Ronald P. Mensink^a^ and Peter J. Joris^a^

^a^Department of Nutrition and Movement Sciences, NUTRIM School for Nutrition and Translational Research in Metabolism, Maastricht University Medical Center, PO Box 616, 6200 MD, Maastricht, the Netherlands.

Corresponding author: Peter J. Joris ([p.joris@maastrichtuniversity.nl](mailto:p.joris@maastrichtuniversity.nl))

Assessed for eligibility (*n* = 23)

Excluded (*n* = 3)

♦  Fasting serum triacylglycerol concentrations > 2.2 mmol/L (*n* = 3)

Discontinued intervention (*n* = 2)

♦ Personal reasons (*n* = 2; sequence CBA and CAB)

Allocated to intervention (*n* = 20)

♦ Received allocated intervention (*n* = 20)

♦ Did not receive allocated intervention (*n* = 0)

Analysed (*n* = 18)

## Allocation

## Analysis

## Follow-Up

Randomized (*n* = 20)

## Enrollment

♦ Sequence ABC (*n* = 2) ♦ Sequence BCA (*n* = 4)

♦ Sequence ACB (*n* = 3) ♦ Sequence CAB (*n* = 4)

♦ Sequence BAC (*n* = 3) ♦ Sequence CBA (*n* = 4)

**Supplemental Fig. 1** Consort flow diagram of the process through the phases of this randomized controlled trial; A: high-fat meal; B: high-carbohydrate meal; C: high-protein meal.
