# Supplementary material for: Dietary macronutrients do not differently affect postprandial vascular endothelial function in apparently healthy overweight and slightly obese men
Source: Eur J Nutr. 2020 Jul 29;60(3):1443–51. doi: 10.1007/s00394-020-02340-y (PMC7987601; doi:10.1007/s00394-020-02340-y)
Supplement: Supplementary file 2 — Supplementary file2 (PDF 183 kb) [file 394_2020_2340_MOESM2_ESM.pdf]

Dietary macronutrients do not differently affect postprandial vascular endothelial function in apparently healthy overweight and slightly obese men

European Journal of Nutrition

Ellen T.H.C. Smeets[a], Ronald P. Mensink[a] and Peter J. Joris[a]

[a]Department of Nutrition and Movement Sciences, NUTRIM School for Nutrition and Translational Research in Metabolism, Maastricht University Medical Center, PO Box 616, 6200 MD, Maastricht, the Netherlands.

Corresponding author: Peter J. Joris (p.joris@maastrichtuniversity.nl)

Supplemental Table 1 The composition of the high-fat, high-carbohydrate and high-protein meal.

	**High-fat**	**High-carbohydrate**	**High-protein**
Energy, kcal	953	953	953
Fat, En% (g)	52.3 (55.3)	9.6 (10.2)	10.6 (11.3)
Saturated Fat, En% (g)	31.3 (33.1)	3.2 (3.4)	3.8 (4.0)
Carbohydrates, En% (g)	39.2 (93.5)	81.5 (194.3)	51.5 (122.7)
Protein, En% (g)	8.0 (19.2)	8.6 (20.4)	36.9 (87.9)
Water, g	297	262	115
