# Supplementary material for: Dietary macronutrients do not differently affect postprandial vascular endothelial function in apparently healthy overweight and slightly obese men
Source: Eur J Nutr. 2020 Jul 29;60(3):1443–51. doi: 10.1007/s00394-020-02340-y (PMC7987601; doi:10.1007/s00394-020-02340-y)
Supplement: Supplementary file 3 — Supplementary file3 (PDF 197 kb) [file 394_2020_2340_MOESM3_ESM.pdf]

Dietary macronutrients do not differently affect postprandial vascular endothelial function in apparently healthy overweight and slightly obese men

European Journal of Nutrition

Ellen T.H.C. Smeets[a], Ronald P. Mensink[a] and Peter J. Joris[a]

[a]Department of Nutrition and Movement Sciences, NUTRIM School for Nutrition and Translational Research in Metabolism, Maastricht University Medical Center, PO Box 616, 6200 MD, Maastricht, the Netherlands.

Corresponding author: Peter J. Joris (p.joris@maastrichtuniversity.nl)

Supplemental Table 2 Fasting total cholesterol, HDL-cholesterol, LDL-cholesterol and hsCRP values of the overweight and slightly obese men who completed the study[a].

Variables	High-fat[b]	High-carbohydrate	High-protein
Total cholesterol (mmol/L)	5.12 ± 0.83	5.25 ± 0.90	5.31 ± 0.86
HDL-cholesterol (mmol/L)	1.20 ± 0.28	1.18 ± 0.26	1.20 ± 0.26
LDL-cholesterol (mmol/L)	3.35 ± 0.78	3.45 ± 0.73	3.55 ± 0.79
hsCRP (mg/L)	1.87 ± 2.25	1.88 ± 1.73	1.52 ± 1.20

[a] HDL: High-density lipoprotein; LDL: low-density lipoprotein; hsCRP; high sensitive C-reactive protein.

[b] Values are means ± SD.
